# Association between a novel Dietary Index for Gut Microbiota and periodontitis: a cross-sectional study

**DOI:** 10.3389/fnut.2026.1714913

**Published:** 2026-01-16

**Authors:** Xinlian Zhang, Xia Lv, Li Zhang, Tingting Jia, Sainan Zhao

**Affiliations:** Department of Stomatology, Affiliated Hospital of Shandong University of Traditional Chinese Medicine, Jinan, Shandong, China

**Keywords:** Dietary Index for Gut Microbiota (DI-GM), periodontitis, body mass index (BMI), NHANES, cross-sectional study

## Abstract

**Background:**

The gut microbiota and periodontitis have attracted increasing research interest. The Dietary Index for Gut Microbiota (DI-GM), a novel metric for assessing gut microbiome diversity, has not yet been investigated in relation to periodontitis.

**Methods:**

This cross-sectional study analyzed data from the 2009–2014 National Health and Nutrition Examination Survey including 9,978 participants aged 30–80 years who had periodontal examination records. Participants were categorized into two groups: no periodontitis (*n* = 4,879) and periodontitis (mild, moderate, or severe; *n* = 5,099). The DI-GM was calculated using dietary recall data, incorporating both beneficial and unfavorable components for gut microbiota. Multivariable logistic regression was applied to examine the association between DI-GM and periodontitis, with body mass index (BMI) evaluated as a potential mediator. Secondary analyses included subgroup evaluations, restricted cubic spline (RCS) modeling, and multivariable imputation.

**Results:**

A higher DI-GM score was inversely associated with periodontitis (odd ratio [OR] = 0.94, 95% confidence interval [CI]: 0.91–0.97). Similarly, a higher beneficial microbiota score was linked to a lower prevalence of periodontitis (OR = 0.90, 95% CI: 0.87–0.94). After adjustment, DI-GM remained inversely associated with moderate (OR = 0.94, 95% CI: 0.91–0.97) and severe periodontitis (OR = 0.89, 95% CI: 0.85–0.94; both *p* < 0.001). Likewise, higher beneficial microbiota scores correlated with reduced odds of moderate (OR = 0.91, 95% CI: 0.87–0.95) and severe periodontitis (OR = 0.84, 95% CI: 0.79–0.90; all *p* < 0.001). The RCS model indicated a linear association between DI-GM and periodontitis. BMI showed a significant mediating effect (4.9, 95% CI: 0.96–11.05%; *p* = 0.014).

**Conclusion:**

The newly proposed DI-GM demonstrated an inverse association with the prevalence of periodontitis, with BMI acting as a significant mediator in this relationship.

## 1 Introduction

Periodontitis is a chronic inflammatory condition primarily driven by dysbiotic bacterial communities. Its progression can result in severe consequences, including tooth exfoliation, alveolar bone resorption, and eventual edentulism (1). In 2021, severe periodontitis affected more than one billion individuals worldwide, with an age-standardized prevalence of 12.5% (2). As a major contributor to the global health burden, periodontitis not only compromises oral health and quality of life but is also closely associated with systemic inflammation and multiple comorbidities, underscoring the need for effective preventive and management strategies (3). Therefore, there is an urgent need to explore effective prevention and management strategies to address the prevalence of periodontitis.

Given this background, emerging evidence suggests a critical link between gut microbiota and periodontitis, emphasizing the interconnectedness of oral and systemic health through the oral–gut axis (4, 5). Previous research showed that masticatory function played a vital role in maintaining oral and gut microbial homeostasis and supporting nutrient absorption, particularly in geriatric nutritional management. Moreover, compromised mastication was shown to directly reduce gut microbial diversity and exacerbate systemic inflammation (6).

Dietary intake, intestinal microbiota, and host physiology constitute a fundamental triad underlying systemic homeostasis. This triad functions through key mechanisms, including immune regulation and metabolic balance (7). Different dietary patterns exert distinct effects on the gut microbiota (8). High-fat diets disrupt gut microbiota, reducing SCFAs and impairing gut barrier, which triggers TLR4/NF-κB-mediated inflammation, promoting obesity and diabetes (9). Conversely, plant-based dietary patterns have been associated with increased microbial diversity and enrichment of beneficial taxa (e.g., *Faecalibacterium, Bifidobacterium*), thereby promoting metabolic health and reducing systemic inflammation lth (10). These findings suggest that dietary interventions targeting the gut microbiota represent a novel and promising therapeutic strategy for improving systemic health outcomes, including oral health.

Building on this evidence, Kase et al. conducted a systematic review of 106 adult studies and identified 14 key dietary components influencing gut microbiota composition. Based on these findings, the researchers developed the Dietary Index for Gut Microbiota (DI-GM), a tool designed to assess diet quality according to its association with gut microbiota health (11). Notably, studies have reported associations between DI-GM and several chronic conditions, including depression (12), diabetes (13) and sleep disorders (14). However, little is known about the potential association between DI-GM and periodontitis.

In addition, obesity—characterized by chronic energy imbalance and adipose tissue remodeling—is recognized as a major contributor to systemic inflammation and metabolic dysregulation (15). Previous research has established associations between obesity and periodontitis (16), as well as between obesity and gut microbiota imbalances (17). Therefore, it is plausible to hypothesize that dietary patterns supporting a healthy gut microbiota may reduce the risk of periodontitis by alleviating obesity-related inflammation and metabolic dysfunction.

Building on these observations, the present study investigates the associations between DI-GM indices and the prevalence of periodontitis among participants of the National Health and Nutrition Examination Survey (NHANES), with particular attention to the potential mediating role of body mass index (BMI). By elucidating these relationships, this study aims to enhance understanding of the oral–gut axis and its implications for dietary strategies aimed at preventing periodontitis.

## 2 Methods

### 2.1 Data sources

Data for this cross-sectional investigation were obtained from three consecutive NHANES cycles (2009–2014). NHANES conducted by the National Center for Health Statistics (NCHS), employs a complex, stratified, multistage probability cluster sampling design to evaluate the health and nutritional status of the civilian, noninstitutionalized U.S. population. The survey protocol was approved by the NCHS Research Ethics Review Board, and all participants provided written informed consent prior to data collection. This secondary analysis qualified for institutional review board exemption. Detailed sampling methodology and data access procedures are available at http://www.cdc.gov/nchs/nhanes.htm. The study design and reporting adhered to the Strengthening the Reporting of Observational Studies in Epidemiology (STROBE) guidelines for observational studies.

### 2.2 Study design and population

**Figure 1:**
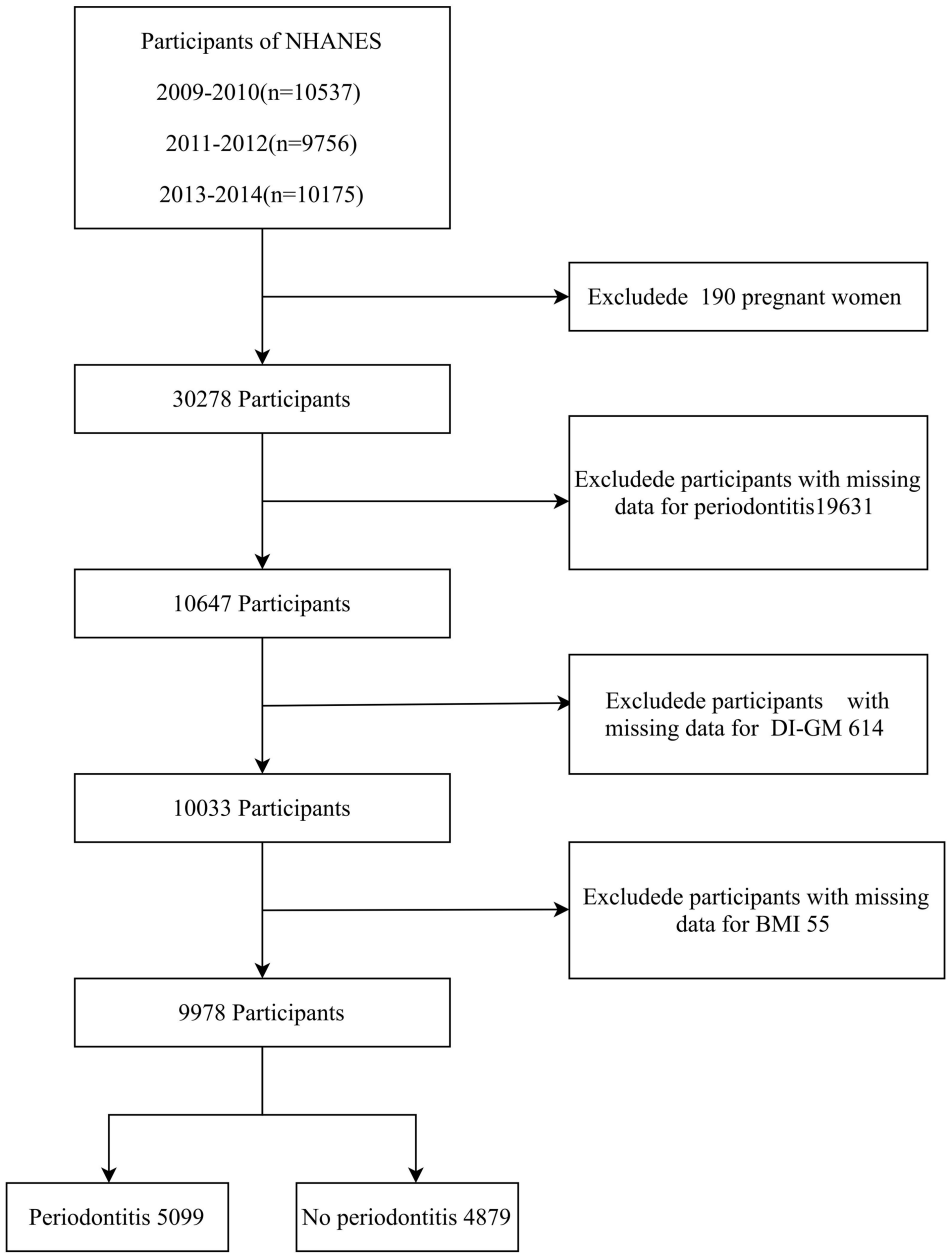
Flow chart of the screening of participants. NHANES, National Health and Nutrition Examination Survey; DI-GM, Dietary Index for Gut Microbiota; BMI, body mass index.

A total of 30,468 participants from the 2009–2014 NHANES data cycles were initially included in the study, as periodontitis data were available only for these cycles. Pregnant women were excluded (*n* = 190). Additional exclusions were applied for missing periodontitis data (*n* = 19,631), incomplete DI-GM component data (*n* = 614), and missing BMI data (*n* = 55). The final cross-sectional analysis included 9,978 participants. The enrollment process is illustrated in Figure 1.

### 2.3 Diagnosis of periodontitis

During the 2009–2014 NHANES cycles, periodontal examinations were conducted at six sites per tooth (mesiobuccal, midbuccal, distobuccal, mesiolingual, midlingual, and distolingual) for up to 28 teeth, following standard periodontal assessment protocols. Two parameters were recorded: clinical attachment loss (AL) and probing depth (PD) (18). Periodontitis was defined according to the classification jointly established by the Centers for Disease Control and Prevention and the American Academy of Periodontology. Mild periodontitis was defined as ≥2 interproximal sites with AL ≥ 3 mm and ≥2 interproximal sites with PD ≥ 4 mm (not on the same tooth), or ≥1 site with PD ≥ 5 mm. Moderate periodontitis was defined as ≥2 interproximal sites with AL ≥ 4 mm (not on the same tooth), or ≥2 interproximal sites with PD ≥ 5 mm (not on the same tooth). Severe periodontitis was defined as ≥2 interproximal sites with AL ≥ 6 mm (not on the same tooth) and ≥1 interproximal site with PD ≥ 5 mm (19). Participants were categorized as having periodontitis (“yes”) if they met any of the above severity criteria (mild, moderate, or severe) or as periodontally healthy (“no”) if none were met.

### 2.4 Development of the DI-GM

Following the standardized scoring criteria proposed by Kase et al., 14 specific food items and nutrients were identified as core components of the DI-GM. Beneficial components included avocado, broccoli, chickpeas, coffee, cranberries, fermented dairy, dietary fiber, green tea (data unavailable because NHANES did not specify tea varieties), soybeans, and whole grains. Adverse components included red meat, processed meat, refined grains, and high-fat diets (11). The DI-GM was calculated using dietary recall data from the 2009–2014 NHANES cycles. For beneficial dietary components, participants received a score of 1 if their intake was greater than or equal to the sex-specific median, and 0 if it was below the median. For unfavorable components, a score of 0 was assigned if consumption was greater than or equal to the sex-specific median (or ≥40% of total energy intake for high-fat diets), and 1 if below the respective threshold. The composite DI-GM score was calculated as the arithmetic sum of all component scores, ranging from 0 (lowest) to 13 (highest), with higher scores indicating closer adherence to the beneficial dietary pattern. This composite score consisted of two parts: beneficial components (score range: 0–9) and unfavorable components (score range: 0–4). For analysis, DI-GM scores were categorized into quartiles (0–3, 4, 5, and ≥6) based on their population distribution.

### 2.5 Covariates

The selection of confounding variables was guided by prior published evidence and clinical relevance. Covariates included age, sex, race/ethnicity, marital status, education level, poverty income ratio (PIR), physical activity level, smoking status, alcohol consumption, diabetes, and hypertension (16, 20, 21). Age was treated as a continuous variable in logistic regression analyses and descriptive statistics, but categorized into three groups for subgroup analyses: 30–44 years, 45–59 years, and 60–80 years. Race/ethnicity was classified as non-Hispanic White, non-Hispanic Black, Mexican American, or other races. Marital status was categorized as married, living with a partner, or living alone. Educational level was divided into three categories: less than 9 years, 9–12 years, and more than 12 years. Based on U.S. government guidelines, family income was categorized into three groups according to PIR: low (PIR ≤ 1.3), medium (PIR > 1.3–3.5), and high (PIR > 3.5). Physical activity was assessed using self-reported exercise intensity, duration, and frequency to calculate weekly metabolic equivalent of task (MET)-minutes. They were classified into three levels according to the International Physical Activity Questionnaire scoring criteria: low (<600 MET-min/week), moderate (600–3,000 MET-min/week), and high (≥3,000 MET-min/week) (22). Smoking status was categorized as never, former, or current smoker, based on responses to two questions: “Have you smoked at least 100 cigarettes in your life?” and “Do you smoke now?” Alcohol consumption was classified into three categories: Never drinkers – individuals who had consumed fewer than 12 drinks in their lifetime; Former drinkers – those who reported consuming at least 12 drinks in a single year but not in the past year, or who abstained during the past year despite previous lifetime consumption of ≥12 drinks; Current drinkers – individuals who consumed at least 12 drinks annually. Prevalent diseases (diabetes and hypertension) were determined based on self-reported physician diagnoses in the questionnaire. BMI was calculated as weight in kilograms (kg) divided by height in meters squared (m^2^).

### 2.6 Statistical analysis

This study involved a secondary analysis of publicly available datasets from NHANES. Continuous variables were expressed as mean ± standard deviation (SD), and categorical variables were presented as proportions (%). Between-group comparisons of continuous variables were conducted using independent-samples t-tests (parametric) or Mann–Whitney U tests (nonparametric), depending on distributional normality. Categorical variables were analyzed using chi-square (χ^2^) tests or Fisher’s exact tests, as appropriate.

For all analyses, covariates had less than 8% missing data. To address missingness while preserving statistical power and minimizing bias, multiple imputation by chained equations (MICE) was applied with five imputations using the mice package in R. All subsequent analyses were performed using the imputed datasets.

Multivariable logistic regression models were constructed to examine the association between DI-GM and periodontitis, including analyses stratified by disease severity. Results were expressed as adjusted odds ratios (ORs) with corresponding 95% confidence intervals (CIs). Model I was unadjusted. Model II was adjusted for age, sex, race/ethnicity, marital status, education level, and PIR. Model III was additionally adjusted for smoking status, alcohol consumption, and physical activity. Model IV was fully adjusted, incorporating Model III covariates plus diabetes and hypertension.

Restricted cubic spline (RCS) regression with four knots positioned at the 5th, 35th, 65th, and 95th percentiles of DI-GM values was applied to evaluate potential nonlinear associations between DI-GM and periodontitis. To further explore underlying mechanisms, the potential mediating role of BMI in the relationship between DI-GM and periodontitis was examined. Mediation effects were assessed using three complementary methods: The Sobel test for parametric significance testing; Nonparametric bootstrapping with 1,000 resamples to estimate confidence intervals; and The quasi-Bayesian Monte Carlo method (1,000 simulations) with normal approximation for robust inference.

### 2.7 Sensitivity analyses: subgroup and multimodel logistic regression

Subgroup analyses were conducted according to age, sex, race/ethnicity, marital status, education level, PIR, smoking status, alcohol consumption, physical activity, diabetes status, and hypertension status. Additionally, multimodel logistic regression analysis was performed using the original dataset after excluding observations with missing values.

All statistical analyses were performed using R software, version 4.2.2 (http://www.R-project.org, The R Foundation for Statistical Computing, Vienna, Austria;) and Free Statistics software, version 2.1.1 (Beijing, China; http://www.clinicalscientists.cn/freestatistics). A two-tailed *p* value < 0.05 was considered statistically significant.

## 3 Results

### 3.1 Characteristics of the participants

A total of 9,978 participants aged 30–80 years were included in the analysis. The overall prevalence of periodontitis was 51.1%. Demographic and clinical characteristics of participants, stratified by periodontitis status, are presented in Table 1. Compared with periodontally healthy participants, those with periodontitis were generally older, included a higher proportion of males, had lower socioeconomic status and educational attainment, engaged in less physical activity, were more likely to be current smokers, and exhibited a higher prevalence of cardiometabolic comorbidities. They also demonstrated lower DI-GM scores and higher BMI values (all *p* < 0.001). However, no significant difference was observed between the two groups in unfavorable-to-gut-microbiota components (*p* > 0.05).

**Table 1:** Comparisons of characteristics between people with no periodontitis and people with periodontitis.

Variables	Total (*n* = 9,978)	No periodontitis (*n* = 4,879)	Periodontitis (*n* = 5,099)	*p*
Age, Mean ± SD	52.1 ± 14.2	48.5 ± 13.6	55.5 ± 13.9	< 0.001
Sex, *n* (%)				< 0.001
Male	4,961 (49.7)	1978 (40.5)	2,983 (58.5)	
Female	5,017 (50.3)	2,901 (59.5)	2,116 (41.5)	
Race/ethnicity, *n* (%)				< 0.001
Non-Hispanic White	4,384 (43.9)	2,488 (51)	1896 (37.2)	
Non-Hispanic Black	2069 (20.7)	804 (16.5)	1,265 (24.8)	
Mexican American	1,431 (14.3)	516 (10.6)	915 (17.9)	
Other	2094 (21.0)	1,071 (22)	1,023 (20.1)	
Marital status, *n* (%)				< 0.001
Married or living with partners	6,466 (64.8)	3,328 (68.2)	3,138 (61.5)	
Living alone	3,512 (35.2)	1,551 (31.8)	1961 (38.5)	
Education level, years, *n* (%)				< 0.001
<9	953 (9.6)	248 (5.1)	705 (13.8)	
9–12	3,504 (35.1)	1,333 (27.3)	2,171 (42.6)	
>12	5,521 (55.3)	3,298 (67.6)	2,223 (43.6)	
Poverty income ratio, *n* (%)				< 0.001
Low income: PIR ≤ 1.3	2,976 (29.8)	1,102 (22.6)	1874 (36.8)	
Medium income: PIR > 1.3–3.5	3,623 (36.3)	1,617 (33.1)	2006 (39.3)	
High income: PIR > 3.5	3,379 (33.9)	2,160 (44.3)	1,219 (23.9)	
Smoking status, *n* (%)				< 0.001
Never	5,575 (55.9)	3,150 (64.6)	2,425 (47.6)	
Former	2,525 (25.3)	1,121 (23)	1,404 (27.5)	
Current	1878 (18.8)	608 (12.5)	1,270 (24.9)	
Alcohol status, *n* (%)				< 0.001
Never	1,336 (13.4)	630 (12.9)	706 (13.8)	
Former	1735 (17.4)	659 (13.5)	1,076 (21.1)	
Now	6,907 (69.2)	3,590 (73.6)	3,317 (65.1)	
Physical activity MET-min/week, *n* (%)				< 0.001
<600	3,973 (39.8)	1853 (38)	2,120 (41.6)	
600–3,000	3,177 (31.8)	1705 (34.9)	1,472 (28.9)	
≥3,000	2,828 (28.3)	1,321 (27.1)	1,507 (29.6)	
Diabetes, *n* (%)				< 0.001
No	8,730 (87.5)	4,471 (91.6)	4,259 (83.5)	
Yes	1,248 (12.5)	408 (8.4)	840 (16.5)	
Hypertension, *n* (%)				< 0.001
No	6,957 (69.7)	3,648 (74.8)	3,309 (64.9)	
Yes	3,021 (30.3)	1,231 (25.2)	1790 (35.1)	
DI_GM_score, Mean ± SD	4.7 ± 1.5	4.8 ± 1.6	4.6 ± 1.5	< 0.001
DI_GM_score.cut, *n* (%)				< 0.001
0–3	2,219 (22.2)	1,011 (20.7)	1,208 (23.7)	
4	2,413 (24.2)	1,086 (22.3)	1,327 (26)	
5	2,377 (23.8)	1,142 (23.4)	1,235 (24.2)	
≥6	2,969 (29.8)	1,640 (33.6)	1,329 (26.1)	
Beneficial to gut microbiota, Mean ± SD	2.3 ± 1.2	2.5 ± 1.3	2.2 ± 1.2	< 0.001
Unfavorable to gut microbiota, Mean ± SD	2.4 ± 1.0	2.4 ± 1.0	2.4 ± 1.0	0.321
BMI, Mean ± SD	29.4 ± 6.7	29.1 ± 6.7	29.6 ± 6.7	< 0.001

SD, standard deviation; DI-GM, Dietary Index for Gut Microbiota; PIR, poverty income ratio; MET, Metabolic Equivalent of Task; BMI, body mass index.

### 3.2 DI-GM–periodontitis association

Table 2 presents the results of the multivariable logistic regression analysis assessing the association between DI-GM and periodontitis after adjustment for potential confounding variables. Each one-point increase in DI-GM was associated with a 10% lower prevalence of periodontitis (OR = 0.90, 95% CI: 0.88–0.92, *p* < 0.001). After full adjustment for confounders (Table 2, Model IV), the association remained significant (OR = 0.94, 95% CI: 0.91–0.97, *p* < 0.001). When DI-GM was analyzed as a categorical variable, participants with DI-GM ≥ 6 had a significantly lower prevalence of periodontitis compared with those in the lowest DI-GM group (OR = 0.68, 95% CI: 0.61–0.76, *p* < 0.001). This association persisted after full adjustment (OR = 0.79, 95% CI: 0.69–0.90, *p* < 0.001; Table 2, Model IV). Furthermore, the score for components beneficial to gut microbiota showed an inverse association with periodontitis (OR = 0.90, 95% CI: 0.87–0.94, *p* < 0.001), whereas components unfavorable to gut microbiota were not significantly associated with periodontitis risk.

**Table 2:** Association between DI-GM and the risk of periodontitis.

Characteristics	Model 1		Model 2		Model 3		Model 4	
	OR (95% CI)	*p* value	OR (95% CI)	*p* value	OR (95% CI)	*p* value	OR (95% CI)	*p* value
DI_GM	0.9 (0.88 ~ 0.92)	<0.001	0.93 (0.9 ~ 0.96)	<0.001	0.94 (0.91 ~ 0.96)	<0.001	0.94 (0.91 ~ 0.97)	<0.001
DI-GM group								
0–3	Ref		Ref		Ref		Ref	
4	1.02 (0.91 ~ 1.15)	0.705	1.05 (0.92 ~ 1.19)	0.49	1.05 (0.92 ~ 1.19)	0.493	1.05 (0.92 ~ 1.2)	0.453
5	0.91 (0.81 ~ 1.02)	0.092	0.96 (0.84 ~ 1.09)	0.526	0.96 (0.85 ~ 1.1)	0.591	0.97 (0.85 ~ 1.11)	0.635
≥6	0.68 (0.61 ~ 0.76)	<0.001	0.76 (0.67 ~ 0.86)	<0.001	0.79 (0.69 ~ 0.89)	<0.001	0.79 (0.69 ~ 0.9)	<0.001
Beneficial to gut microbiota	0.84 (0.81 ~ 0.86)	<0.001	0.9 (0.87 ~ 0.93)	<0.001	0.91 (0.87 ~ 0.94)	<0.001	0.9 (0.87 ~ 0.94)	<0.001
Unfavorable to gut microbiota	1.02 (0.98 ~ 1.06)	0.321	0.99 (0.95 ~ 1.03)	0.646	0.99 (0.95 ~ 1.04)	0.82	1 (0.96 ~ 1.04)	0.964

OR, Odd Ratio; CI, Confidence interval; Ref, reference; DI-GM, Dietary Index for Gut Microbiota; PIR, poverty income ratio.

Model 1: crude model.

Model 2: age, sex, race/ethnicity, marital status, education level, PIR.

Model 3: Model 2 + smoking status, alcohol status, physical activity.

Model 4: Model 3 + Diabetes, Hypertension.

**Figure 2:**
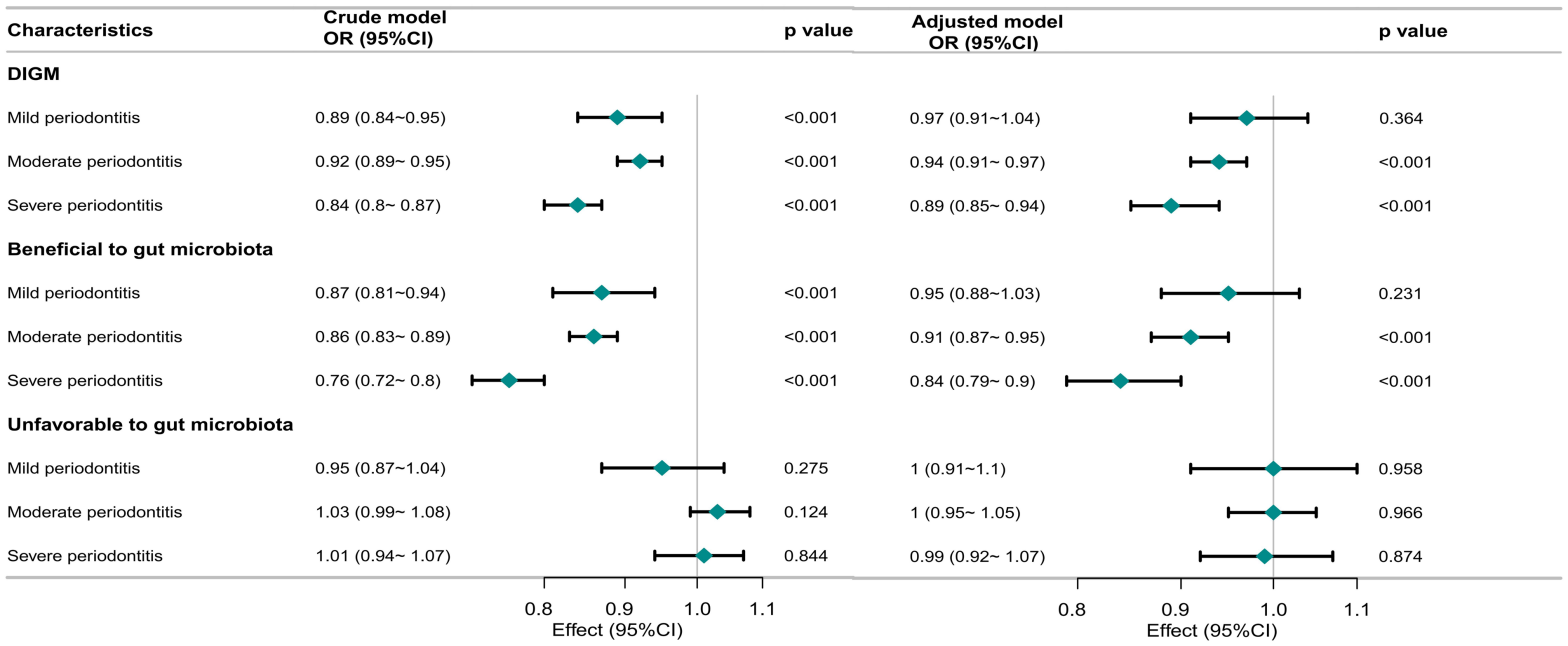
Association of DI-GM with periodontitis severity in participants. The crude model was not adjusted for any covariates, while the adjusted model was adjusted for age, sex, race/ethnicity, marital status, education level, PIR, smoking status, alcohol status, physical activity, Diabetes, hypertension. OR, odd ratio; CI, confidence interval; DI-GM, Dietary Index for Gut Microbiota.

Figure 2 illustrates the dose–response relationship between DI-GM and periodontitis severity across different clinical categories. Multivariable-adjusted models demonstrated an inverse relationship between DI-GM and both moderate (OR = 0.94, 95% CI: 0.91–0.97, *p* < 0.001) and severe periodontitis (OR = 0.89, 95% CI: 0.85–0.94, *p* < 0.001). Higher levels of beneficial-to-gut-microbiota components were also associated with a lower prevalence of moderate (OR = 0.91, 95% CI: 0.87–0.95, *p* < 0.001) and severe periodontitis (OR = 0.84, 95% CI: 0.79–0.90, *p* < 0.001). In contrast, no significant association was observed between unfavorable-to-gut-microbiota components and periodontitis risk.

**Figure 3:**
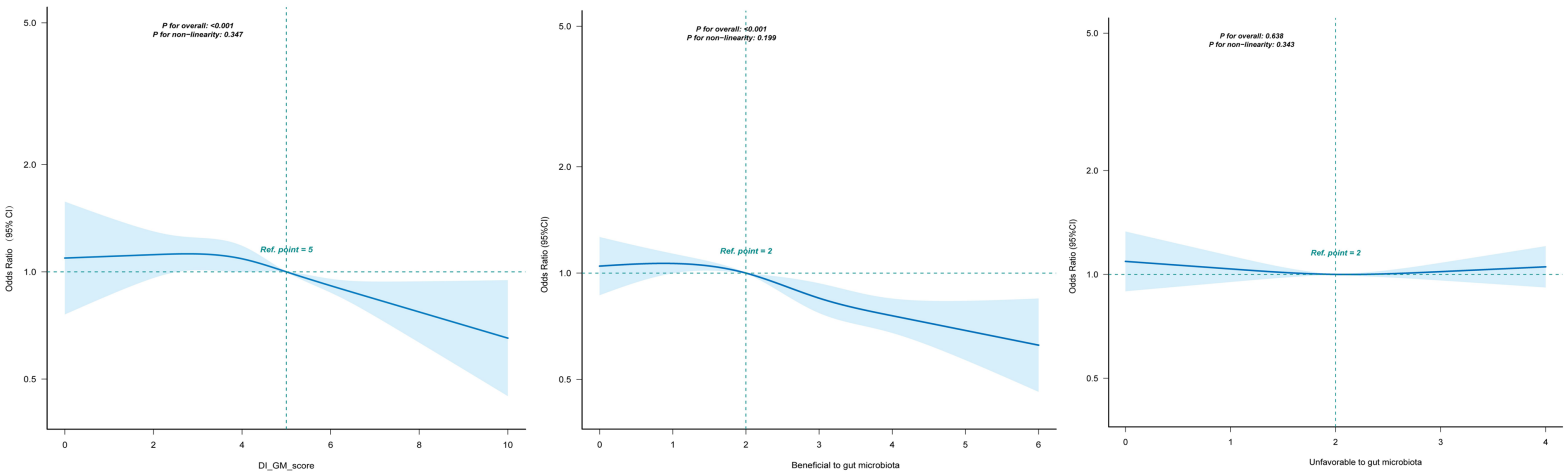
Association between DI-GM and periodontitis in participants by RCS. Solid and dashed lines represent the predicted value and 95% confidence intervals. They were adjusted for age, sex, race/ethnicity, marital status, education level, PIR, smoking status, alcohol status, physical activity, diabetes, hypertension. CI, confidence interval; DI-GM, Dietary Index for Gut Microbiota; PIR, poverty income ratio; RCS, restricted cubic spline.

As shown in Figure 3, DI-GM was linearly associated with periodontitis (*P* for nonlinearity = 0.347). Similarly, beneficial-to-gut-microbiota components exhibited a linear dose–response relationship with periodontitis (*P* for nonlinearity = 0.199). In contrast, unfavorable-to-gut-microbiota components showed no significant association with periodontitis (*p* > 0.05). Overall, an inverse relationship was observed between DI-GM levels and periodontitis risk, with higher DI-GM scores associated with progressively lower odds of disease.

### 3.3 Mediation and supplementary analyses

**Figure 4:**
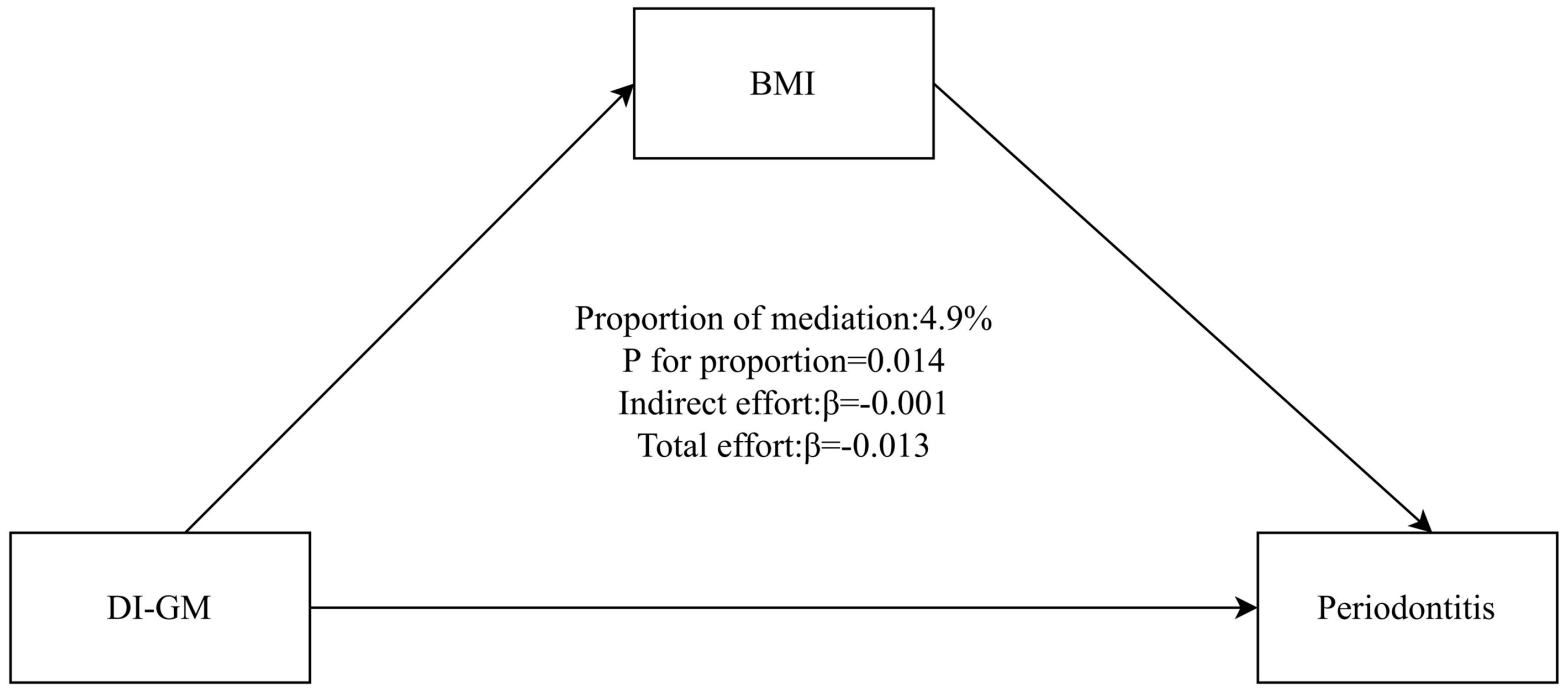
Mediation analysis of BMI in the association between DI-GM and periodontitis. The model adjusted for age, sex, race/ethnicity, marital status, education level, PIR, smoking status, alcohol status, physical activity, diabetes, hypertension. BMI, body mass index; DI-GM, Dietary Index for Gut Microbiota; PIR, poverty income ratio.

Figure 4 illustrates that BMI mediated the association between DI-GM and periodontitis. The mediation effect of BMI was statistically significant, accounting for 4.9% of the total effect (95% CI: 0.96–11.05%, *p* = 0.014).

Comprehensive sensitivity analyses were conducted using multiple inferential models to assess the robustness of the primary findings and to evaluate potential variations in study conclusions. Subgroup analyses were also performed, including stratified analyses across various demographic and clinical subgroups. For continuous variables, categorization was performed based on established clinical cutoffs prior to conducting interaction analyses. DI-GM consistently demonstrated a protective association with periodontitis across all subgroups, with no significant interaction effects observed (*p* > 0.05; Supplementary Figure 1). Moreover, after excluding participants with missing covariate data (n = 1,296), subsequent analyses reconfirmed the robustness of the DI-GM–periodontitis association, indicating the stability of the findings (Supplementary Table 2). In multimodel logistic regression analyses, participants with DI-GM ≥ 6 had a significantly lower prevalence of periodontitis (crude model: OR = 0.69, 95% CI: 0.61–0.78, *p* < 0.001; adjusted model: OR = 0.80, 95% CI: 0.70–0.92, *p* = 0.002).

## 4 Discussion

This large cross-sectional study found that higher DI-GM scores were robustly associated with a lower prevalence of periodontitis, exhibiting a dose–response relationship independent of major confounders. Notably, this inverse association extended to periodontitis severity and was partially mediated by BMI. This finding underscores that dietary intervention may reduce periodontitis risk by optimizing systemic metabolism through the gut microbiota, illustrating the diet-microbiota-periodontitis axis.

Periodontitis remains a leading cause of tooth loss among adults and substantially affects quality of life and overall health. It has a multifactorial etiology, in which subgingival biofilm triggers host inflammatory and immune responses, ultimately resulting in irreversible periodontal tissue destruction (23). Periodontitis has been associated with reduced *α*-diversity in the gut microbiota (24). Fecal samples from individuals with periodontitis show increased relative abundances of Bacteroides, *Faecalibacterium*, *Fusobacterium*, and *Lachnospiraceae*, and decreased abundance of *Lactobacillus* (25). Inflammatory bowel disease is also linked to periodontitis, and the microbial species implicated in this association include *Fusobacterium nucleatum, Campylobacter rectus, and Campylobacter concisus* (26). The literature supports a bidirectional relationship between intestinal inflammation and periodontitis, wherein each condition appears to influence the onset and progression of the other. Metabolites produced by the gut microbiota can contribute to the pathogenesis of periodontitis through the oral–gut axis (27). Within this axis, pathobiont-responsive Th17 cells, regulated by the intestinal microbiome, have been shown to induce periodontal inflammation (28). Growing evidence indicates that intestinal microbial communities play a central role in regulating bone metabolism via the intestine-to-alveolar bone signaling pathway. Gut microbiota and their metabolites may be translocated through systemic circulation to the alveolar bone, directly influencing periodontal tissue homeostasis and stability (29).

The protective effect of microbiota-supportive diets likely operates through modulation of systemic inflammation. Diets rich in components beneficial to gut microbiota—such as dietary fiber and polyphenols—enhance the production of SCFAs by commensal bacteria (30). SCFAs exert potent systemic anti-inflammatory effects, potentially mitigating the inflammatory cascade that drives periodontal tissue destruction (31). Conversely, dysbiotic diets high in saturated fats and refined sugars promote the enrichment of proinflammatory taxa (e.g., *Firmicutes*), reduce beneficial genera (e.g., *Bifidobacterium*), and foster a state of metabolic inflammation conducive to the pathogenesis of periodontitis (9). This mechanistic framework aligns with established evidence that periodontitis results from a dysregulated host inflammatory response to subgingival biofilm, leading to irreversible tissue damage and concurrent alterations in gut microbial composition and diversity (23, 24).

Our findings are consistent with prior research reporting inverse associations between Mediterranean or plant-based dietary patterns and periodontitis (32, 33). This concordance is biologically plausible, as high DI-GM scores inherently emphasize key components of these dietary patterns—namely fruits, vegetables, legumes, and whole grains—which are known to promote microbial homeostasis and attenuate inflammation (34). Supporting evidence comes from a systematic review of 14 studies (1998–2018) demonstrating that both dietary vitamin C intake and serum concentrations exhibit inverse associations with periodontitis prevalence and severity (35). Our study carries potential clinical implications, suggesting periodontitis patients increase intake of high-DIGM foods while limiting low-DIGM foods. This finding supports that dietary counseling aimed at promoting microbiota-supportive foods—particularly those rich in fiber and polyphenols—could serve as a valuable adjunct to conventional periodontal therapy by modulating systemic inflammatory pathways and improving treatment outcomes.

This study introduces a novel application of the DI-GM index, a tool that employs 14 specific food components to quantify a gut-friendly diet. The index demonstrates a robust correlation with biomarkers of gut microbiome diversity, enabling precise identification of dietary patterns that enhance microbial diversity. Furthermore, its emphasis on specific foods, rather than broad categories, enhances its clinical applicability. Unlike traditional scores (e.g., HEI-2015, MED), the DI-GM maintains comparable overall diet quality assessment while providing a more comprehensive evaluation of diet-microbiome interactions by incorporating microbial features like SCFA production.

The significant mediation by BMI underscores a plausible biological pathway linking diet, gut microbiota, and periodontitis. Substantial evidence indicates that obesity contributes to the pathogenesis of periodontitis, partly through adipose tissue secretion of proinflammatory cytokines (e.g., tumor necrosis factor-alpha, interleukin-6) and adipokines that stimulate osteoclast activity and promote tissue destruction (36, 37). Gut microbiota dysbiosis is a pivotal factor in the development of obesity and its associated metabolic inflammation (38). The present observation that BMI mediates the association between DI-GM and periodontitis suggests that dietary patterns fostering a healthy gut microbiota may improve periodontal health partly by exerting beneficial effects on body weight and its related inflammatory milieu.

The strengths of this study include its large, nationally representative NHANES sample and the use of standardized, validated periodontal assessment protocols. However, several limitations merit consideration. The cross-sectional design precludes causal inference. Dietary data were derived from self-reported 24-h recalls, which are subject to recall bias and may not accurately represent habitual intake. Although comprehensive adjustments were made for potential confounders, residual confounding from unmeasured variables cannot be excluded. Furthermore, the DI-GM constructed for this analysis lacked the “green tea” component of the original index due to unavailable data in NHANES. Given that green tea polyphenols possess documented anti-inflammatory properties, this omission may have attenuated the observed inverse association. Future prospective cohort studies are warranted to establish causality, and longitudinal intervention trials should further evaluate the efficacy of dietary strategies targeting the gut microbiota for the prevention and management of periodontitis.

## 5 Conclusion

Using data from a nationally representative sample, this study demonstrated that greater adherence to the DI-GM was associated with a lower prevalence of periodontitis, particularly in moderate and severe cases. The observed association was partially mediated by BMI. Collectively, these findings suggest that diets supporting gut microbial balance, as quantified by the DI-GM, may contribute to reduced periodontitis risk partially through obesity-related pathways.

## Data Availability

The datasets presented in this study can be found in online repositories. The names of the repository/repositories and accession number(s) can be found at: https://www.cdc.gov/nchs/nhanes/index.htm.

## Supplementary material

SUPPLEMENTARY FIGURE 1 Forest plot showing the association between DI-GM and the risk of periodontitis in different subgroups. Except for the stratification component itself, each stratification factor was adjusted for all other variables (age, sex, race/ethnicity, marital status, education level, PIR, smoking status, alcohol status, physical activity, Diabetes, Hypertension). DI-GM, Dietary Index for Gut Microbiota; MET, Metabolic Equivalent of Task; OR, odds ratio; 95% CI, 95% confidence interval.
