# Supplementary material for: Association between a novel Dietary Index for Gut Microbiota and periodontitis: a cross-sectional study
Source: Front Nutr. 2026 Jan 16;13:1714913. doi: 10.3389/fnut.2026.1714913 (PMC12857318; doi:10.3389/fnut.2026.1714913)
Supplement: Supplementary file 2 [file Table_1.docx]

**Table 1**. Comparisons of characteristics between people with no periodontitis and people with periodontitis ( original data )

| Variables | Total  (n = 9978) | No periodontitis (n = 4879) | Periodontitis (n = 5099) | p |
| --- | --- | --- | --- | --- |
| Age, Mean ± SD | 52.1 ± 14.2 | 48.5 ± 13.6 | 55.5 ± 13.9 | < 0.001 |
| Sex, n (%) |  |  |  | < 0.001 |
| Male | 4961 (49.7) | 1978 (40.5) | 2983 (58.5) |  |
| Female | 5017 (50.3) | 2901 (59.5) | 2116 (41.5) |  |
| Race/ethnicity, n (%) |  |  |  | < 0.001 |
| Non-Hispanic White | 4384 (43.9) | 2488 (51) | 1896 (37.2) |  |
| Non-Hispanic Black | 2069 (20.7) | 804 (16.5) | 1265 (24.8) |  |
| Mexican American | 1431 (14.3) | 516 (10.6) | 915 (17.9) |  |
| Other | 2094 (21.0) | 1071 (22) | 1023 (20.1) |  |
| Marital status, n (%) |  |  |  | < 0.001 |
| Married or living with partners | 6462 (64.8) | 3326 (68.2) | 3136 (61.6) |  |
| Living alone | 3510 (35.2) | 1551 (31.8) | 1959 (38.4) |  |
| Education level,years, n (%) |  |  |  | < 0.001 |
| ＜9 | 951 ( 9.5) | 248 (5.1) | 703 (13.8) |  |
| 9-12 | 3501 (35.1) | 1333 (27.3) | 2168 (42.6) |  |
| ＞12 | 5515 (55.3) | 3294 (67.6) | 2221 (43.6) |  |
| Poverty income ratio, n (%) |  |  |  | < 0.001 |
| Low income:≤1.3 | 2706 (29.5) | 1008 (22.2) | 1698 (36.6) |  |
| Medium income:  >1.3-3.5 | 3305 (36.0) | 1492 (32.9) | 1813 (39) |  |
| High income: >3.5 | 3175 (34.6) | 2041 (44.9) | 1134 (24.4) |  |
| Smoking status, n (%) |  |  |  | < 0.001 |
| Never | 5573 (55.9) | 3150 (64.6) | 2423 (47.5) |  |
| Former | 2524 (25.3) | 1121 (23) | 1403 (27.5) |  |
| Current | 1878 (18.8) | 608 (12.5) | 1270 (24.9) |  |
| Alcohol status, n (%) |  |  |  | < 0.001 |
| Never | 1236 (13.1) | 580 (12.5) | 656 (13.6) |  |
| Former | 1647 (17.4) | 631 (13.6) | 1016 (21.1) |  |
| Now | 6561 (69.5) | 3412 (73.8) | 3149 (65.3) |  |
| Physical activity MET-min/week,n(%) |  |  |  | < 0.001 |
| ＜600 | 3973 (39.8) | 1853 (38) | 2120 (41.6) |  |
| 600-3000 | 3177 (31.8) | 1705 (34.9) | 1472 (28.9) |  |
| ≥3000 | 2828 (28.3) | 1321 (27.1) | 1507 (29.6) |  |
| Diabetes, n (%) |  |  |  | < 0.001 |
| No | 8724 (87.5) | 4468 (91.6) | 4256 (83.5) |  |
| Yes | 1248 (12.5) | 408 (8.4) | 840 (16.5) |  |
| Hypertension, n (%) |  |  |  | < 0.001 |
| No | 6942 (69.8) | 3640 (74.8) | 3302 (65) |  |
| Yes | 3008 (30.2) | 1229 (25.2) | 1779 (35) |  |
| DI_GM_score, Mean ± SD | 4.7 ± 1.5 | 4.8 ± 1.6 | 4.6 ± 1.5 | < 0.001 |
| DI_GM_score.cut, n (%) |  |  |  | < 0.001 |
| 0-3 | 2219 (22.2) | 1011 (20.7) | 1208 (23.7) |  |
| 4 | 2413 (24.2) | 1086 (22.3) | 1327 (26) |  |
| 5 | 2377 (23.8) | 1142 (23.4) | 1235 (24.2) |  |
| ≥6 | 2969 (29.8) | 1640 (33.6) | 1329 (26.1) |  |
| Beneficial to gut microbiota, Mean ± SD | 2.3 ± 1.2 | 2.5 ± 1.3 | 2.2 ± 1.2 | < 0.001 |
| Unfavorable to gut microbiota, Mean ± SD | 2.4 ± 1.0 | 2.4 ± 1.0 | 2.4 ± 1.0 | 0.321 |
| BMI, Mean ± SD | 29.4 ± 6.7 | 29.1 ± 6.7 | 29.6 ± 6.7 | < 0.001 |

Numbers that do not add up to 100% are attributable to missing data.

Abbreviations: SD, standard deviation; DI-GM, dietary index for gut microbiota; PIR, poverty income ratio; MET, Metabolic Equivalent of Task; BMI, body mass index.
