# Supplementary material for: Association between a novel Dietary Index for Gut Microbiota and periodontitis: a cross-sectional study
Source: Front Nutr. 2026 Jan 16;13:1714913. doi: 10.3389/fnut.2026.1714913 (PMC12857318; doi:10.3389/fnut.2026.1714913)
Supplement: Supplementary file 3 [file Table_2.docx]

**Table 2** Association between DI-GM and the risk of periodontitis.

| Characteristics | Model 1 | | Model 2 | | Model 3 | | Model 4 | |
| --- | --- | --- | --- | --- | --- | --- | --- | --- |
|  | OR (95% CI) | p value | OR (95% CI) | p value | OR (95% CI) | p value | OR (95% CI) | p value |
| DI_GM | 0.91(0.88~0.93) | <0.001 | 0.94 (0.91~0.97) | <0.001 | 0.94 (0.91~0.98) | 0.001 | 0.95 (0.92~0.98) | 0.001 |
| DI-GM group |  |  |  |  |  |  |  |  |
| 0-3 | Ref |  | Ref |  | Ref |  | Ref |  |
| 4 | 1.03 (0.91~1.17) | 0.652 | 1.06 (0.92~1.22) | 0.420 | 1.05 (0.91~1.2) | 0.522 | 1.05 (0.91~1.21) | 0.474 |
| 5 | 0.94 (0.83~1.07) | 0.365 | 1.02 (0.88~1.17) | 0.833 | 1 (0.87~1.16) | 0.967 | 1.01(0.87~1.16) | 0.917 |
| ≥6 | 0.69 (0.61~0.78) | <0.001 | 0.78 (0.68~0.90) | <0.001 | 0.8 (0.7~0.92) | 0.001 | 0.8(0.7~0.92) | 0.002 |
| Trend.test |  | <0.001 |  | <0.001 |  | 0.001 |  | 0.001 |
| Beneficial to gut microbiota | 0.84(0.82~0.87) | <0.001 | 0.91 (0.87~0.94) | <0.001 | 0.91 (0.88~0.95) | <0.001 | 0.91(0.88~0.95) | <0.001 |
| Unfavorable to gut microbiota | 1.02(0.98~1.06) | 0.317 | 1(0.96~1.05) | 0.938 | 1 (0.96~1.05) | 0.874 | 1.01(0.96~1.06) | 0.735 |

The multimodel logistic regression analysis was conducted based on the original data with all missing values deleted.

OR: Odd Ratio, CI: Confidence interval, Ref: reference, DI-GM: dietary index for gut microbiota, PIR: poverty income ratio.

Model 1:crude model

Model 2:age, sex, race/ethnicity, marital status, education level, PIR

Model 3:Model 2+smoking status, alcohol status, physical activity

Model4:Model 3+Diabetes, Hypertension
